# Kinetics of alkoxysilanes hydrolysis: An empirical approach

**DOI:** 10.1038/s41598-019-54095-0

**Published:** 2019-11-26

**Authors:** Ahmed A. Issa, Marwa El-Azazy, Adriaan S. Luyt

**Affiliations:** 1 Department of Chemistry and Earth sciences, CAS, Qatar University, Doha, Qatar; 2 Center of Advanced Materials, Qatar University, Doha, Qatar

**Keywords:** Chemistry, Materials science

## Abstract

Alkoxysilanes and organoalkoxysilanes are primary materials in several industries, e.g. coating, anti-corrosion treatment, fabrication of stationary phase for chromatography, and coupling agents. The hydrolytic polycondensation reactions and final product can be controlled by adjusting the hydrolysis reaction, which was investigated under a variety of conditions, such as different alkoxysilanes, solvents, and catalysts by using gas chromatography. The hydrolysis rate of alkoxysilanes shows a dependence on the alkoxysilane structure (especially the organic attachments), solvent properties, and the catalyst dissociation constant and solubility. Some of the alkoxysilanes exhibit intramolecular catalysis. Hydrogen bonding plays an important role in the enhancement of the hydrolysis reaction, as well as the dipole moment of the alkoxysilanes, especially in acetonitrile. There is a relationship between the experimentally calculated polarity by the Taft equation and the reactivity, but it shows different responses depending on the solvent. It was found that negative and positive charges are respectively accumulated in the transition state in alkaline and acidic media. The reaction mechanisms are somewhat different from those previously suggested. Finally, it was found that enthalpy–entropy compensation (EEC) effect and isokinetic relationships (IKR) are exhibited during the hydrolysis of CTES in different solvents and catalysts; therefore, the reaction has a linear free energy relationship (LFER).

## Introduction

**Figure 1:**
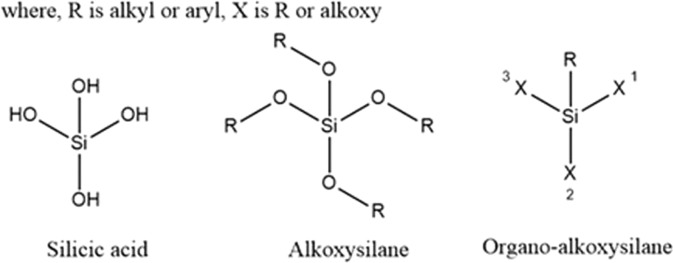
Chemical structures of silicic acid, alkoxysilanes (AS), and organoalkoxysilanes (OAS).

Alkoxysilanes (AS) and organoalkoxysilanes (OAS), with chemical structures, shown in Fig. 1, are the starting materials in several industrial applications. OAS is identified as mono-, di-, tri-, and tetra-alkoxysilanes according to the number of leaving groups. Silicic acid (Si(OH)_4_) can be assumed as the parent compound for all the AS. Though there is a difference between the silanes, AS and OAS, these names will be used interchangeably in this paper to refer to the same compound.

The reactivity of these compounds is controlled by the hydrolysis reaction, represented in Eq. 1. In excess water, the hydrolysis is a pseudo-first order reaction. The rate of the reaction is equal to *k*[*AS*], where k is the rate constant (s^−1^) and [AS] is the concentration of the AS. The concentration of AS at any time can be estimated using the integrated form of the first order equation shown in Eq. 2.

1 $$\equiv \,Si-O-R+{H}_{2}O\mathop{\to }\limits^{k}\equiv Si-OH+R-OH$$

2 $$[AS]={[AS]}_{0}\,{e}^{-kt}$$

Therefore, the determination of the consumed AS and the reaction extent depend on the rate constant (k). According to the transition state theory, the quasi-equilibrium constant (${K}^{\ddagger }$) between the activated complex and reactants is proportional to the Gibbs free energy ($\Delta {G}^{\ddagger }$) (Eq. 3).

3 $$\Delta {G}^{\ddagger }=-RTln({K}^{\ddagger })$$

The rate constant is related to $\Delta {G}^{\ddagger }$ (Eq. 4), where, R, T, $\,{\boldsymbol{\kappa }}$, and h are universal gas constant, absolute temperature, Boltzmann constant, and Planck’s constant, respectively. Therefore, the rate constant is controlled by ($\Delta {G}^{\ddagger }$), and any change in the free energy will lead to a change in the rate constant.

4 $$k=\frac{{\boldsymbol{\kappa }}T}{h}\,{e}^{\frac{-\Delta {G}^{\ddagger }}{RT}}$$

Several approaches were used to determine the rate constant, depending on the change in $\Delta {G}^{\ddagger }$; (i) Linear free energy relationship (LFER), which is an empirical approach, e.g. Hammett and Taft equations^1^; (ii) Computational chemistry using different methods^2–7^; (iii) The structure reactivity relationship (SAR)/quantitative structure activity relationship (QSAR); (iv) Computational approach that depends on different methods, LFER, SAR, and perturbation molecular orbital (PMO) to estimate the hydrolysis rate of carboxylic and phosphate esters in different solvents^8,9^.

Currently the computational chemistry is limited to very small molecules and a highly restricted reaction environment^2–7^, which is not the situation in the AS reaction. In general, SAR and QSAR require enough and precise data to construct correlations between the descriptors and the rate constant, which are not available. Starting with the LFER approach and the Hammett equation (Eq. 5), Akerman *et al*.^1^ determined the *ρ* values of different phenoxy derivatives of triethylphenoxysilanes in acidic and alkaline media. They found that *ρ* equals 0.533 and 1.743 for acidic and alkaline media, respectively. Since that time, there were no real trials to find a correlation between the absolute rate constants and the reaction conditions, except one trial to find a correlation between the rate constant, and the proton [H^+^] and hydroxide [OH^−^] concentrations.

5 $$\mathrm{ln}(\frac{k}{{k}_{o}})=\rho \sigma $$

where, *k* and *k*_*o*_ are the rate constants of the substituted and the parent compounds, respectively, *ρ* is a constant that represents the reaction medium effect in the case of constant substitution, *σ* is a constant that represents the substituent effect in case of constant reaction medium effect. Equation 6 was derived from this.

6 $$\Delta {G}^{\ddagger }=\Delta {G}_{o}^{\ddagger }-2.3\,RT\rho \sigma $$

This contribution is a combination of steric, resonance, and polar effects, considering the AS structure and keeping in mind the Hammett equation (Eq. 5), where *σ* represents the effect of a substituent on the reaction rate constant. Taft *et al*.^10^ determined the polar effect by using Eq. 7 suggested in Ingold’s work^11^, where they reported that the hydrolysis rate is influenced by steric and resonance effects in an acidic medium, but by steric, resonance, and polarity effects in an alkaline medium.

7 $${\sigma }^{\ast }=[\,log{(k/{k}_{o})}_{B}-{[\log (k/{k}_{o}))}_{A}]$$

where σ* is the polar effect, *k* and *k*_*o*_ are the rate constants of the substituted and parent esters, respectively. A and B refer to acidic and alkaline media. The steric hindrance (E_s_) can be evaluated by Eq. 8.

8 $${E}_{s}=log{(k/{k}_{o})}_{A}\,$$

In this work, the effect of the structures of AS on the hydrolysis rate constant in acidic, alkaline, and fluoride of protic (methanol), moderate polar aprotic (acetonitrile), and non-polar aprotic solvents (dioxane) media, in addition to the effect of the reaction medium on the hydrolysis rate, were investigated. Since the Hammett equation is applicable to aromatic compounds, especially meta- and para- substituents, the separation of polarity and steric factors by the Taft equations will be a suitable approach to study the effect of both on the AS hydrolysis. Depending on the hydrolysis investigation under a variety of conditions, different mechanisms are suggested for the investigation of hydrolysis under a variety of conditions, and this can be a subject for further studies. In line with the work done by Linert^12,13^ on isokinetic relationships (IKR), the effect of the reaction medium on different aspects of the hydrolysis of 3-cyanopropyltriethoxysilane (CTES) was investigated.

## Materials and Methods

### Materials

The used AS (Table 1) was purchased from Millipore-Sigma, USA. Ethyl acetate, tetrahydrofuran (THF), and ammonium hydroxide were from Sigma-Aldrich, methanol from Lichrosolv, while acetonitrile and n-butanol were from BDH. Dimethyl formamide (DMF), dioxane, and acetic acid were purchased from Riedel-de*-*Haën, Koch-light laboratory, and Hipersolv, respectively. All the solvents were used without further purification. The water contents were determined using Karl Fischer (KF) reagents (Generator Solution 1613 and Vessel Solutions 1612 from GFS Chemicals, USA). The properties of the used solvents are tabulated in Table 1.

**Table 1:** Abbreviations, names, structures and calculated properties (pK_a_^16^, partition coefficients^18^, α and β values^18^, molecular volume (V)^17^, and logP^17^) of used OAS.

Organoalkoxysilanes (OAS)		pK_a_			logP (water, organic solvent)			(α and β of silane)		V	LogP
Abbreviation	Name/structure				Methanol	Acetonitrile	Dioxane	α	β		
OTES		0.5	0.8	1.6	5.22	6.22	6	0	0.287	1146	4.7
CTES		0.4	0.5	1.2	2.67	3.36	2.88	0.033	0.275 (O) 0.438 (N)	908	2.76
PTES		0.1	1.3	1.3	2.71	3.50	3.33	0	0.28 (O) 0.489 (Si)	887	3.47
ISBTES		0	0	1.3	3.46	4.15	3.78	0	0.287(O)	856	2.95
STES		0.9	0.9	1	2.98	3.94	3.73	0.131(S)	0.282(O) 0.235(S)	895	3.63
ATES		0.2 N (10)	0.6	1.6	0.58	1.28	1.01	0.035(N)	0.285(O) 0.735(N)	873	2.12
DMDES		−1.1	−0.7		1.82	1.47	1.95	0	0.376(O)	628	2.18
DPDES		2.5	2.6		4.92	5.6	6.2	0	0.365(O)	970	4.91
TMES		−2.6			2.57	2.45	2.65	0	0.466(O)	563	1.97
TEOS		0.8	0.9	1.1 1.2	2.96	3.2	3.09	0	0.198(O)	799	2.25

### Preparation of working solutions

The kinetic studies were conducted through a batch technique; the work was divided into two groups of batches: (1) The first group (room temperature), in which the temperature was not controlled, while the compositions of the reaction medium and silanes were systematically changed; (2) For the second group the temperature was controlled within limits by using a heat block, in addition to changing the reaction medium compositions. Each batch contained eight samples for the same AS due to a limitation in GC auto injector sample positions. We were careful to minimize the experimental errors as much as possible by standardizing the procedure for all the AS and using only one AS in each batch and decrease the GC run time to collect more points over 24 hours for each AS. After collecting the chromatograms by using GC as described in the next section, the data was used to obtain a rate constant through integration. These values, in addition to other reported and theoretically calculated values, were used in the other calculations as described in Section 2.4.

The first group of batches (that are discussed in Sections 3.1 to 3.3) were prepared as follows to study the effect of the combination of solvents, catalysts, and AS structure:

- In this experiment, three solvents were selected, protic (methanol), dipolar aprotic (acetonitrile), and nonpolar aprotic (dioxane). All three solvents are completely miscible with water. The water content was adjusted to 3.5 M with deionized water, and this we called adjusted solvents.
- For each AS, three vials were prepared as follows for each solvent: 1600 μL from the adjusted solvent plus 40 μL from deionized water for the spontaneous conditions, while for the catalytic conditions we used 40 µL of one of the following catalysts: glacial acetic acid, 1 M NaF, and 6 M ammonium hydroxide. The run started with the addition of 30 μL AS to the vial.

The second group of batches (that are discussed in Sections 3.4 and 3.5) were prepared as follows to determine the thermodynamic parameters and to study the enthalpy-entropy compensation (EEC):

The effect of solvents on the activation energy, pre-exponential factor, and thermodynamic quantities of the activated complex: The water content for each solvent (Table S1) was determined by KF, and then adjusted to 2 M by adding deionized water. The run started by adding 40 µL of CTES to 1500 µL of the water-adjusted solvent at different temperatures (RT, 40, 60, 90 °C), and the initial time (t_i_) was recorded. The samples were prepared in well-closed 2.0 mL vials (not GC vials). The prepared samples were heated in heating blocks from Stuart, SHB1000KIT, UK. At injection, the sample was manually removed from the heating block, the temperature reduced to room temperature by cooling. The vial was then opened, and the sample immediately injected into the GC, the final time (t_f_) was recorded, and vial was properly closed and again returned to the heating block.

### Characterization techniques

Gas chromatography (GC) (Agilent, 6890N, USA), equipped with a flame ionization detector (FID), split/splitless inlet, and HP-5 column was used throughout the study to separate and determine the reaction components. The used method was described in detail in our previous paper^14^. Briefly, the prepared samples were injected into the GC using the following settings: Inlet at temperature 260 °C with split mode 1:50 ratio, detector (FID) at temperature 300 °C, with hydrogen gas as fuel, zero air as oxidizing gas, and helium gas as makeup gas with flow rates 50, 350 and 35 mL min^−1^, respectively. The helium was used as a carrier gas at a flow rate of 1.2 mL min^−1^. The temperature programme of the oven was as follows: holding the temperature at 40 °C for 5 min, ramping the temperature up to 90 °C at a rate of 5 °C min^−1^, then ramping it up again to 260 °C at a rate of 10 °C min^−1^, and finally holding the temperature at 260 °C for 10 min. Karl Fischer (KF) from Mettler Toledo C20 was used to determine the water content in the reaction medium.

### Source of theoretical data and calculations

After the GC chromatograms were acquired, the area under the peak for the OAS was determined using Chemstation Version B software. A ln(area) *vs*. time (s) graph was then plotted, and the slope of the line is equal to -k_ob_ (observed rate constant). The corrected rate constant (k_corr_) was calculated by dividing the observed rate constant by the catalyst concertation (M). Under spontaneous conditions, the rate constant (k_spon_) is equal to the observed or corrected ones. The polarity (σ*) and the steric effect (E_s_) were calculated using Eqs. 7 and 8, respectively^10^. The polarity of the attached groups were obtained from ref. ^15^. The molecular volume and pK_a_ of ethoxy’s oxygen were calculated by QikProp and Jaguar software, respectively, from Schrodinger LLC^16,17^. The SPARC program from Archem LLC was used to estimate the partition coefficients of OAS between the organic solvents (methanol, acetonitrile, and dioxane) and water, and the hydrogen-bond properties (e.g. alpha and beta)^18^. The thermodynamic quantities of the activated complex were calculated from the activation energies (E_a_) and the pre-exponential factors (A), that resulted from the plotting of the Arrhenius graphs, and according to Eqs. 9 to 11.

9 $$\Delta {H}^{\ddagger }={E}_{a}-RT$$

10 $$\Delta {S}^{\ddagger }=Rln(A)-Rln({k}_{B}T/h)-R$$

11 $$\varDelta {G}^{\ddagger }=\varDelta {H}^{\ddagger }$$

## Results and Discussion

### Effect of different variables

In this paper, the effect of solvent, catalyst, and structure combination on the hydrolysis rate of AS is investigated.

**Figure 2:**
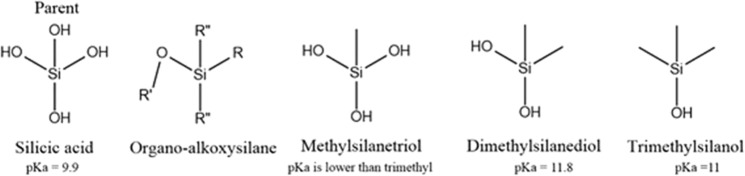
Scheme of the structures of silicic acid (parent), OAS, dimethylsilanetriol, dimethylsilanediol, and trimethylsilanol (hydrolyzed form of TMES)^24^.

As shown in Fig. 2, the AS are formed from an organo group (R), leaving group (R’O^−^), and organo/leaving (R”) groups. The leaving group could be alkoxides (e.g. methoxide, ethoxide) or conjugated bases (e.g. acetate, formate), while the organo-groups could be any organic functional group. The effect of the leaving group is outside the scope of this paper, although it was noticed that the hydrolysis of AS with a conjugated base as leaving group is much faster than in the case of an alkoxide. This could be attributed to the formation of an acid, in which case the reaction becomes autocatalytic. Åkerman *et al*.^1^ reported that the hydrolysis rate constant is proportional to the polarity (σ) of phenoxy derivatives according to the Hammet equation (Eq. 5), while Brinker *et al*.^19^ stated that the hydrolysis rate is related to the pK_a_ of the parent alcohol of the leaving group. The silicic acid has at least two pK_a_ values (9.84 and 13.43) due to their presence as ${\rm{SiO}}{({\rm{OH}})}_{3}^{1-}\,{\rm{and}}\,{{\rm{SiO}}}_{2}{({\rm{OH}})}_{2}^{2-}$ ^20^. Moreover, as the substituents of silicic acid are varied, the pK_a_ values will change, as in the case of DMDES and TMES. This is reflected in their hydrolysis rate constants (Tables 2 and 3). Starting from this point, and utilizing the Hammett, Taft and Grunwald-Winstein approaches and equations, the effect of solvent, catalysts, and structure will be discussed in the next subsections.

**Table 2:** Log (k_spon_) and other calculated parameters of alkoxysilanes in different solvents.

Solvent	Methanol	Acetonitrile	Dioxane	Ethoxy groups	Molecular volume	α	β
Catalyst	NO	NO	NO				
TEOS	−5.85	−5.85	−5.95	4	799	—	—
ISBTES	−5.91	−5.54		3	856	0	0.287(O)
ATES	−3.44	−4.52	−4.61	3	873	0.035 (N)	0.285(O) 0.735(N)
DMDES	−4.03	−8.52	−7.05	2	628	0	0.376(O)
TMES	−4.85	−5.77	−5.29	1	563	0	0.466(O)

**Table 3:** Log(k_corr_) of different OAS in acidic, fluoride and alkaline media in methanol, acetonitrile, and dioxane.

Solvent	Methanol			Acetonitrile			Dioxane		Ethoxy groups
Catalyst	Acetic acid	NaF	NH_4_OH	Acetic acid	NaF	NH_4_OH	Acetic acid	NH_4_OH	
TEOS	−3.262	−4.371	−2.939	−6.452	−4.561	−4.019	−6.092	−3.869	4
OTES	−2.749	−2.620	−3.848	−4.014	−5.579	−7.190	−7.652	−5.427	3
CTES	−3.290	−1.893	−3.354	−4.980	−3.189	−5.111	−7.538	−5.076	3
PTES	−2.999	−2.419	−3.500	−5.292	−3.994	−4.960	−5.573	−4.848	3
ISBTES	−2.953	−2.514	−4.828	−5.197	−5.266	−8.190	−8.652	−5.991	3
STES	−2.783	−2.263	−3.659	−4.801	−3.703	−5.014	−5.698	−4.952	3
ATES	−3.442	−0.512	−2.580	−2.902	−2.032	−3.650	−2.462	−3.830	3
DMDES	−2.430	−2.005	−3.600	−3.205	−4.555	−6.646	−6.009	−5.427	2
DPDES	−2.874	−2.793	−3.389	−5.358	−4.157	−6.076	−6.097	−5.735	2
TMES	−0.795	−0.549	−1.345	−2.749	−3.794	−6.209	−4.611	−4.720	1

#### Spontaneous conditions

**Figure 3:**
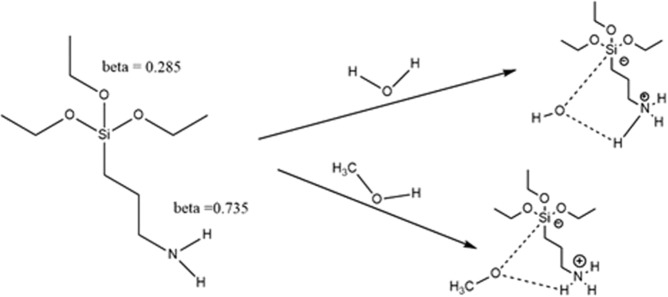
Structural scheme showing the enhancement of the hydrolysis rate of ATES in methanol due to formation, which leads to enhancing its solvation in methanol and then its hydrolysis.

The hydrolysis reactions of TEOS, DMDES, TMES, ATES, and ISBTES in a protic solvent (methanol), moderate polar aprotic solvent (acetonitrile), and a non-polar aprotic solvent (dioxane) were investigated. AS was selected to represent mono-, di-, tri-, and tetra-function silanes, and because their spontaneous hydrolysis rate is relatively high compared to the others. The log(k_spon_) for TEOS in all solvents is almost the same, while the rate constant order is ISBTES < TEOS < TMES < DMDES < ATES in methanol, which is aligned with logP (methanol/water) rather than with the molecular volume or the number of function groups. The orders in acetonitrile and dioxane are similar, but the rate constant values are different, where the order is DMDES < TEOS < TMES < ISBTES < ATES. The rate constants in dioxane is a little bit higher than in acetonitrile, despite the fact that acetonitrile is more polar than dioxane, implying that the activated complex is not polar. In comparison to the other AS, ATES shows a better hydrolysis rate, especially in methanol, due to intramolecular catalysis (Fig. 3 and Table 2).

The solvent can play an important role in enhancing the hydrolysis rate by increasing the solvation of the activated complex. The ionizing power (π*), nucleopholicity (N), and basicity of the solvent control the solvolysis reaction of the activated complex^21^. The ionizing power, however, has little effect on the reaction, which is clear from the free energy of transfer (∆G_trans_) of the hydrolysis of AS from acetonitrile to dioxane (Eq. 12).

12 $${\Delta G}_{trans}=-{\rm{RT}}(\log ({k}_{ace}/{k}_{dio})$$

where k_ace_, and k_dio_ are the hydrolysis rate constants of AS in acetonitrile (ε = 37) and dioxane (ε = 2), respectively. For example, for TMES, ∆G_trans._ = −RT(−5.77 − (−5.29)) = 1.2 kJ. In addition, the hydrolysis rate constants of the AS in methanol is ten times larger than that in acetonitrile and dioxane. This can be attributed to hydrogen bonding and re-esterification. The protic solvent (methanol) also has a good solvation power to anions (hydroxide group)^22^. The hydrolysis rate of most AS under spontaneous conditions is usually extremely slow. However, an approximate prediction of rate can be achieved depending on the molar volume, β, and the calculated logP (using solvent/water) for the targeted AS.

#### Catalytic conditions

Generally, the hydrolysis of AS in an acidic medium is in order of protic > polar aprotic > non-polar aprotic, while in an alkaline medium it is in the order protic > non-polar aprotic > polar aprotic, except in the case of amino alkoxysilane, where the hydrolysis rate in dioxane > acetonitrile > methanol in the case of using acetic acid as a catalyst. At the same time, the hydrolysis of ATES is in the order of methanol > acetonitrile > dioxane in the case of using ammonium hydroxide. However, the hydrolysis rate of AS using NaF as a catalyst in methanol is higher than in acetonitrile. The corrected hydrolysis rate of AS in a fluoride medium seems to be higher than those in alkaline and acidic media, which could be due to the dissociation constants of the catalysts in the different media, or the efficiency of the fluoride ion as a catalyst.

**Figure 4:**
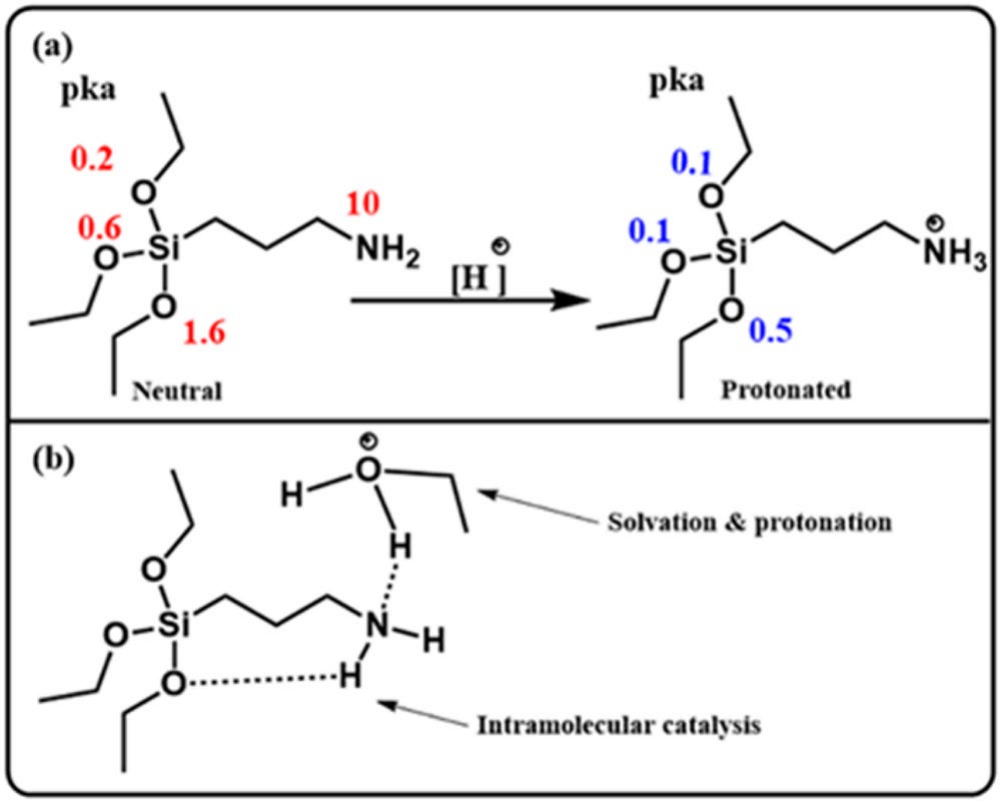
(**a**) Calculated pKa of neutral and protonated amino silane in water by the Jaguar software^16^, and (**b**) the intramolecular catalysis and reaction between the protonated methanol and amino (NH_2_) groups in ATES.

In all the investigated AS, the catalyzed hydrolysis reaction was much faster than the un-catalyzed ones, except in the case of the amino silanes. In the case of using ammonium hydroxide, the observed rate constants (k_obs_) in both cases (catalyzed or non-catalyzed) were almost the same. In the case of using acetic acid in methanol, the rate constant decreased with addition of the catalyst, while in acetonitrile and dioxane, the hydrolysis rate constant increased with addition of acid. The hydrolysis in the fluoride media behaved similar to the other AS (Tables 2 and 3). The inhibition effect of acetic acid can be explained by the protonation of the amino group, where its pK_a_ and β values are respectively 10 and 0.735, which leads to the consumption of a proton in the protonation of the amino group instead of the ethoxy group. Figure 4 shows the intramolecular catalysis in amino silanes, the inhibition effect of the acid, and the formation of non-methoxy monomers.

The hydrolysis of AS is a proton transfer reaction, where water is one of the reactants. An organic solvent is used as a homogenizing agent, and most of the AS are oil soluble, in addition to the existence of a preferential solvation effect. All the above contribute to complicate the prediction of the reaction rate. The influence of the reaction medium composition and the structure effect of AS was investigated through systematic change (Table 3).

#### Effect of partition coefficient

**Figure 5:**
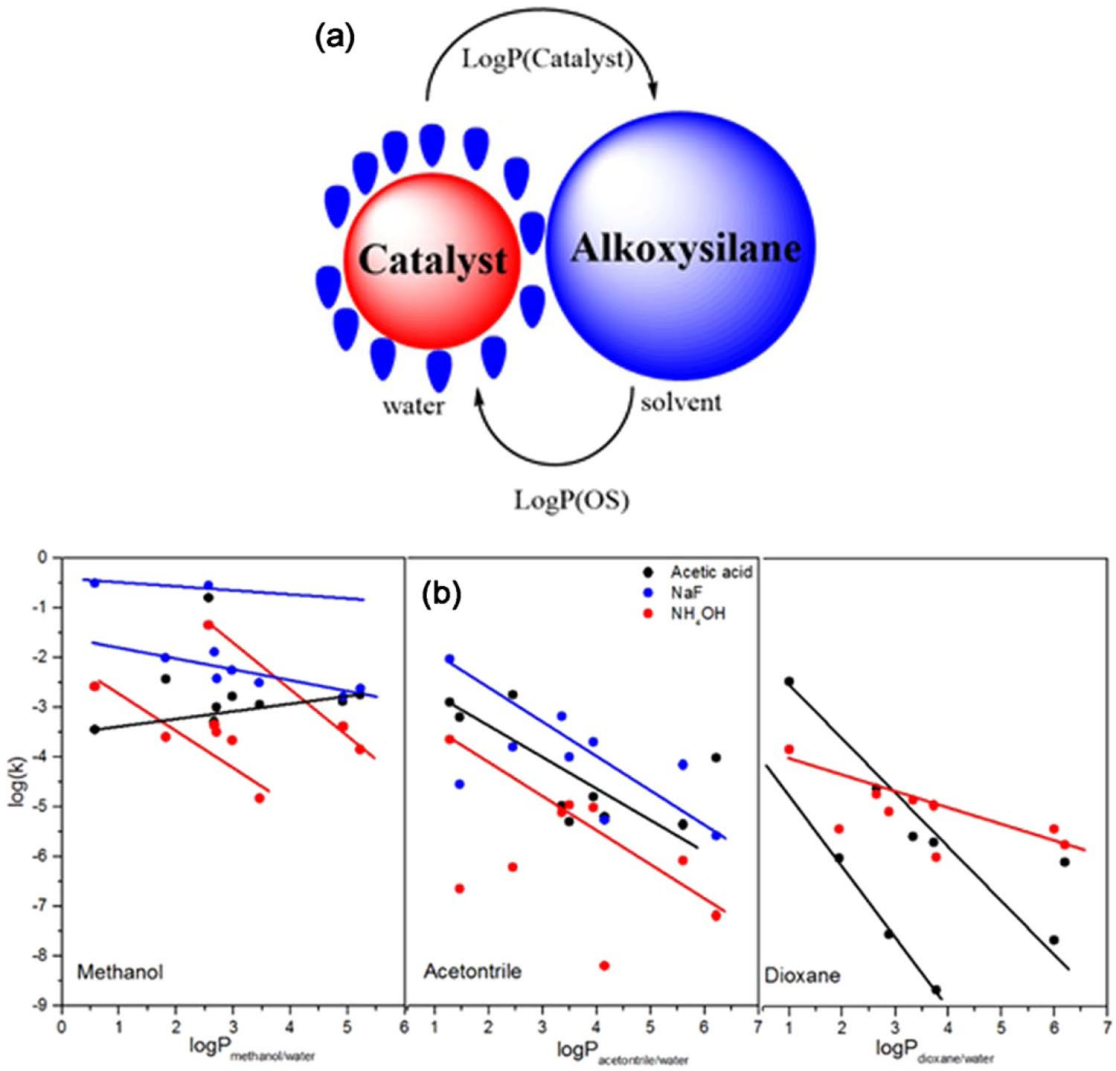
(**a**) Schematic diagram to show the preferential solvation of catalyst and alkoxysilane, and the reactivity of OS vs logP in (b-left) methanol, (b-middle) acetonitrile, and (b-right) dioxane.

Due to the preferential solvation of OAS and the catalyst between the homogenizing agent (organic solvent) and water, the partition coefficient plays an important role in the hydrolysis reaction. Most of the OAS dissolve in the organic zone, and the catalyst in the water zone. The difference in logP between the OAS and the catalyst can be assumed as a resistance gap (Fig. 5(a)). When this gap becomes much smaller, the hydrolysis rate becomes much faster. Figure 5(b) shows that the hydrolysis rate decreases with increasing the logP of the OAS, except when using an acidic medium in methanol, which could be because the protic solvent (methanol) is a very good proton solvator, and hence methanol will transfer the proton to the OAS in which it effectively solvated^22^. The hydrolysis rate increased linearly in an acidic medium in methanol, but it shows an exponential increase in the case of DMDES and TMES. This could be due to a decrease in their molecular volume. The hydrolysis rate in a fluoride medium in methanol (blue lines) slowly decreased, with a deviation in the case of ATES due to intramolecular catalysis, and in the case of TMES due to a small molecular volume. The hydrolysis in an alkaline medium in methanol (red lines) shows two groups of AS, which could be due to the steric effect around the silicon atom. The hydrolysis rates decreased rapidly with increasing the logP, which is probably because the protic solvent is a poor anion solvator and the ammonium hydroxide is a weak base^22^.

The hydrolysis in acidic, fluoride, and alkaline media in acetonitrile is highly scattered, but it is possible to assume that the decreases are in parallel with increasing the logP of OAS. This could be because acetonitrile has weak amphiprotic properties in addition to the fact that it has poor anion and cation solvator effects leading to a general inhibition of the hydrolysis rate. The hydrolysis of OAS decreased rapidly in an acidic medium in dioxane, which could be due to the dissociation constant of acetic acid; when the acetic acid leaves the water zone to the organic zone, it becomes an associated form (confirmed by GC in unpublished work). The hydrolysis of OAS in an alkaline medium decreased slowly with increasing logP of OAS.

#### Effect of AS structures

**Figure 6:**
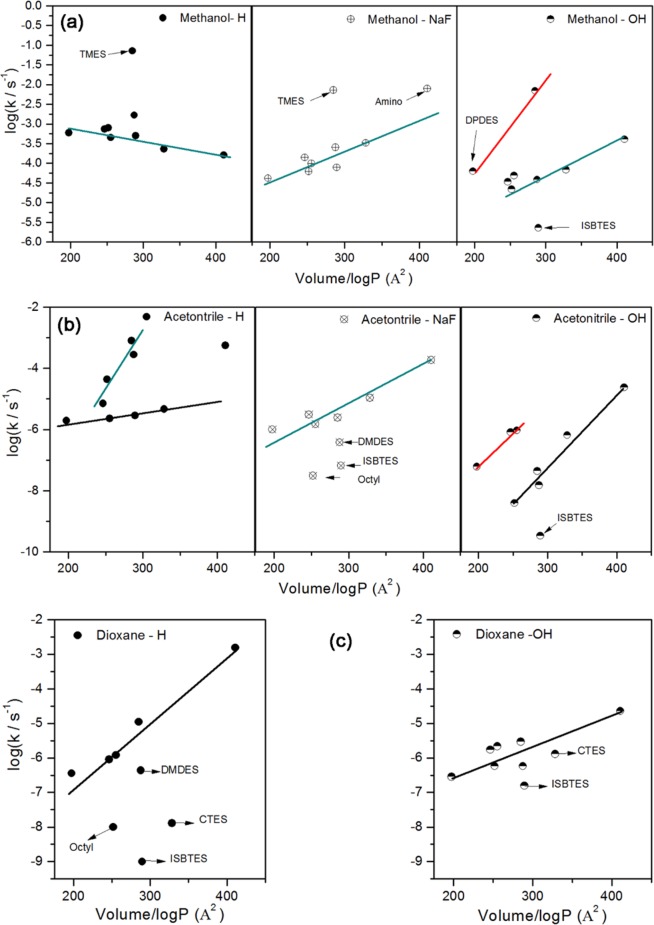
Relation between log(k) and VP ratio of each silane in different solvents and with different catalysts depending on structure.

Figure 6 shows that the reactivity of AS, the relation between log(k_corr_) and the ratio of the molecular volume to partition coefficient (VP) of each AS was plotted for different combinations of solvent and catalyst. In general, the reactivity power, trend, and order for the investigated AS changed with changing the composition of the reaction medium. The structure effect will be discussed in the case of fixing the catalyst and varying of solvent, then fixing the solvent and changing the catalyst.

Using acetic acid in methanol, the reaction rate constant decreased with increasing the volume:logP ratio, with the exception of TMES. This could be due to the molecular volume as explained previously. In acetonitrile, the hydrolysis rate increased with increasing the VP ratio, but as two groups, one which increased more rapidly than the other. This could be the result of the steric effect (represent by volume) of AS by interaction of acetonitrile with AS whose the highest dipole moment, where the acetonitrile has high dielectric constant (37). In an acidic medium in dioxane, the hydrolysis increased with the VP ratio, with some deviations. In fluoride media, the hydrolysis rate increased regularly with increasing the ratio, with some exceptions in acetonitrile due to the hydrophobicity of AS.

In an alkaline medium the hydrolysis rate generally increased with increasing the VP ratio. This probably also depends on the dielectric constant and hydrophobicity of the OAS in acetonitrile.

### Polarity (σ*) and steric (Es) parameters through Taft equation

**Figure 7:**
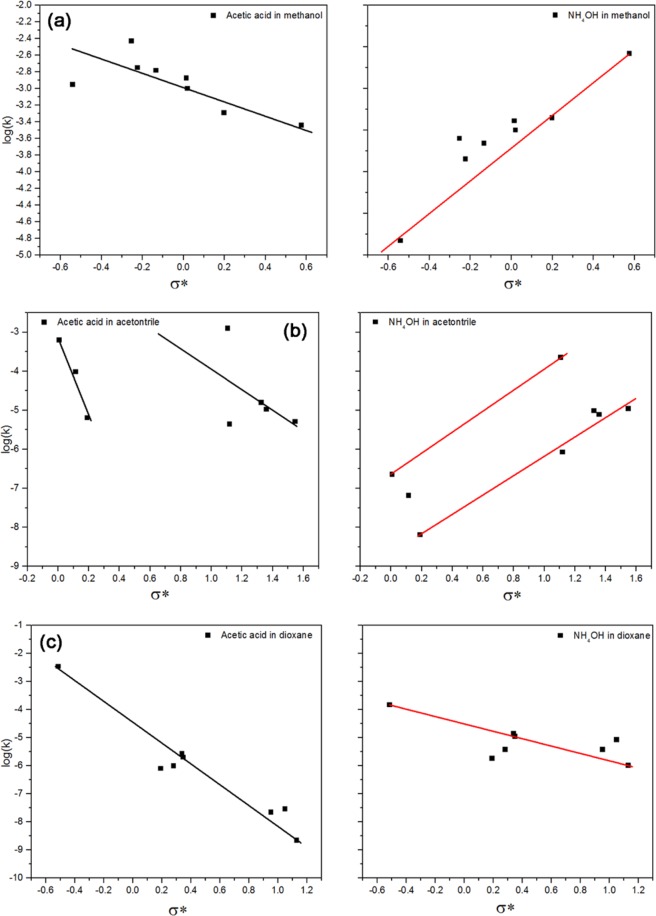
Relation between log(k_corr_) and the calculated σ* in acidic and alkaline media in: (**a**) methanol; (**b**) acetonitrile; (**c**) dioxane.

As already mentioned, the Hammett equation is suitable only for meta- and para-substituted aromatic compounds, while it fails to deal with ortho-aromatic substitution and aliphatic compounds. Taft used the Ingold notification about the hydrolysis of esters in acidic and alkaline media to separate the steric and polarity effects^10^. In this it is assumed that the activated complex is tetrahedral, and that the protons and hydroxide ions are too small to change the steric strain during the transfer from alkaline to acidic media. Based on this assumption, the polarity (σ*) is separated from the steric (Es) effect by Eqs. 7 and 8. Although AS forms a pentavalent activated complex during hydrolysis, the same assumption was used to study the effect of polarity and steric parameters on the hydrolysis of the AS (Table 4). The polarity data in the table is represented in Fig. 7. The steric effect could measure the capability of the solvent to form a shell by electrostatic forces or hydrogen bonding around the alkoxysilanes. For example, OTES is more hydrophobic and has smaller H-bond parameters (α and β) than ATES. ATES has the highest steric effect in methanol, which decreases gradually with decreasing the solvent polarity, while OTES has the lowest (except DMDES) steric effect in methanol, although it has a significant steric value due to its ability to form an H-bond with water and methanol, which increases with decreasing the solvent polarity. In general, the steric effect increases with increasing logP of AS in methanol, while it decreases with increasing the logP in acetonitrile and dioxane, which could be due to the ability of the solvent and AS to expel or attract the water molecules from/to the internal shell.

**Table 4:** Polar and steric parameters of the alkoxysilanes in Table 3 by using the Taft equations and TMES as a reference.

	Methanol		Acetonitrile		Dioxane	
	Polarity	Steric	Polarity	Steric	Polarity	Steric
TEOS	0.356	−2.467	2.405	−3.703	0.951	−1.481
OTES	−0.224	−1.954	0.116	−1.265	0.953	−3.041
CTES	0.198	−2.495	1.359	−2.231	1.049	−2.927
PTES	0.020	−2.204	1.548	−2.543	0.340	−0.962
ISBTES	−0.541	−2.158	0.191	−2.448	1.131	−4.041
STES	−0.133	−1.988	1.325	−2.052	0.349	−1.087
ATES	0.576	−2.647	1.107	−0.153	−0.514	2.149
DMDES	−0.253	−1.635	0.008	−0.456	0.282	−1.398
DPDES	0.014	−2.079	1.119	−2.609	0.192	−1.486
TMES	0.000	0.000	0.000	0.000	0.000	0.000

Figure 7 correlates the calculated polarity and log(k_corr_) of the AS. The slopes of the lines are generally negative in an acidic medium and positive in an alkaline medium, indicating the accumulation of positive charge on the activated complex in an acidic medium, and negative charge in an alkaline medium. The value of the slope shows the effectiveness of the substituent polarity on the reaction center, i.e. the sensitivity of the hydrolysis rate to the polarity of the substituent. In the case of a positively charged activated complex (acidic medium), the sensitivity of the hydrolysis rate to the polarity of the substituent is in the order acetonitrile > dioxane > methanol. In acetonitrile, the plot shows two lines, which may depend on the hydrophobicity and dielectric constant within each series.

For the negatively charged activated complex (in an alkaline medium), the reactivity increased with increasing the electron withdrawing groups in methanol and acetonitrile. The hydrolysis rate generally decreased with increasing the electron withdrawing groups in dioxane, but the points appear as groups on the graph that depend on the logP of AS in dioxane (Fig. 7 (lower panel), where the slope increases with increasing polarity within each group). It can therefore be concluded that the effect of substituent polarity depends on the charges formed on the activated complex. The sensitivity of the hydrolysis to change in the polarity of the substituent depends on the interaction between the AS and the solvent, especially on logP of the solute between the used solvent and water.

**Figure 8:**
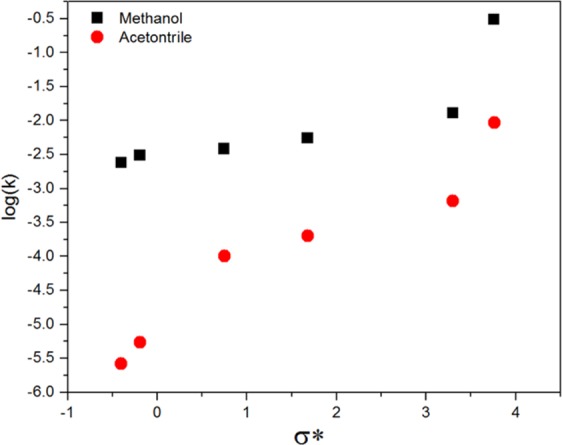
Relation between polarity of triethoxysilane attached groups and hydrolysis rate in fluoride solvents.

Figure 8 shows the direct effect of the polarity of the attached groups on the hydrolysis rate of OAS, where the hydrolysis rate decreased with increasing inducing power (−I) of the electron donating group, while it increased with increasing (+I) of the electron withdrawing group. Although the hydrolysis rate in methanol is higher than that in acetonitrile, the latter has a significant effect on the relation between the hydrolysis rate and the polarity of the attached groups. In addition, the functional group in ATES exists as (NH_3_^+^) and not as amino (NH_2_). The ethoxy group is a very strong electron donating group compared to the alkyl group, so any replacement of exthoxy with alkyl or another electron withdrawing group will lead to electron deficiency on the silicon, and the silicon atoms will become partially positive (δ^+^), which will be targeted by the fluoride ion.

### Effect of the reaction medium and proposed reaction mechanism

**Figure 9:**
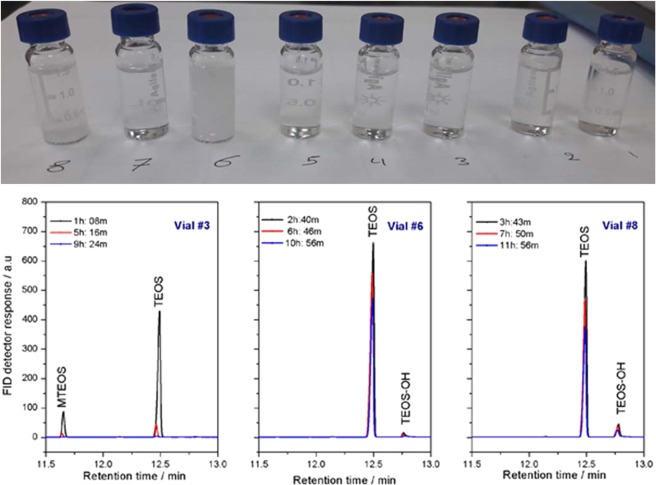
A photo of TEOS hydrolytic polycondensation in different media after one day (top), and chromatograms of TEOS hydrolysis in an alkaline medium in methanol (vial #3), acetonitrile (vial #6), and dioxane (vial #8) at different times (bottom).

The composition of the reaction medium plays an important role in the hydrolysis of AS as explained in the previous sections. It also has a direct effect on the whole hydrolytic polycondensation process. In this section we briefly discuss the hydrolytic polycondensation in acidic and fluoride media. TEOS and its methoxy derivatives disappeared in the alkaline medium in methanol after about five hours, but the hydrolyzed monomers could not be observed from the beginning. The TEOS concentration decreased slowly over time, while the hydrolysis rate in acetonitrile is smaller than in dioxane. However, the formation of the precipitate (silica particles) did not depend on the hydrolysis rates. No precipitate formed in methanol, even after completion of the hydrolysis after five hours. A precipitate was formed in acetonitrile as a suspension and in dioxane as dense particles (Fig. 9 (upper panel)). This confirms our previous observation that the hydrolysis rate in alkaline dioxane is faster than in alkaline acetonitrile, despite the fact that the acetonitrile is more polar than the dioxane. The hydrolysis reaction in acidic and fluoride media was very slow, except in acidic methanol. In this case there are some unanswered questions. How is it possible that the negative charge (OH^−^) attacks the partially negatively charged Si atom? Why does the fluoride, which is also negative and has the same ionic size, not catalyze the reaction in the same way as the hydroxide? Why does the proton, which is positive, not attack the partially negative silicon atom?

In an attempt to understand the mechanism of the catalysts, the area under the peak of the corresponding AS in ethanol was determined (Table 5). Acetonitrile was selected because of its polarity and to avoid the effect of the formation of an H-bond. Ethanol was therefore selected to monitor the hydrolysis rates, because small changes can be easily observed. It is clear that the efficiency of the catalyst depended on the AS dipole moment, where ammonia dominated below a dipole moment of 1.5 D, acetic acid between 1.5 and 3 D, and fluoride above 6 D. This was probably due to the effect of the high dielectric constant of acetonitrile. This confirmed that the catalysts behaved differently toward AS polycondensation, and depended on its organic attached groups. The suggested mechanism is that the OH groups attack the AS from the back to form a pentavalent transition state (TS) in an alkaline medium, while the ethoxy oxygen was rapidly protonated in an acidic medium, after which water attacked the silicon atom in a slow step^2,5^. However, according to the results represented in Table 5 and Fig. 9, it is suggested that the hydroxide catalyzed the hydrolysis reaction by attacking the α-hydrogen atom in the ethoxy group, and then the water attacked the silicon atom as was the case of the proton in an acidic medium. The fluoride ions attacked only the silicon atom to form pentavalent TS.

**Table 5:** The area under the peak of the produced ethanol after hydrolytic polycondensation of AS in acidic, fluoride, and alkaline media in acetonitrile.

AS	H	NaF	OH	Dipole moment
TEOS	44.8	17.3	686	1.25
OTES	161	10	3	1.58
CTES	247	566.2	95.7	6.36
PTES	95	102.9	68.5	3.05
ISBTES	139.8	0	0	1.55
STES	278	289	117.5	2.71
ATES	833	434	110	1.53
DMDES	720	55.1	19.1	1.87
DPDES	68.5	37.1	5	1.64
TMES	380	54.4	14.2	1.73

### Thermodynamic studies of the solvent effect on spontaneous CTES hydrolysis

#### Calculation of thermodynamic quantities for CTES activated complex in non-catalytic solvents

For a closed system, the activation energies ranged between 23.4 to 42.5 kJ mol^−1^, and ∆H^ǂ^ ranged between 20.9 and 40.1 kJ mol^−1^. An interesting observation is that, despite the variation in the activation energies, enthalpies, and entropies, the ∆G^ǂ^ remained very close to 112.2 kJ mol^−1^ for all solvents except methanol and ethyl acetate, for which the ∆G^ǂ^ values were respectively 107.9 and 110.3 kJ mol^−1^. The reaction therefore undergoes enthalpy- and entropy compensation, which we shall discuss in more detail later on.

**Figure 10:**
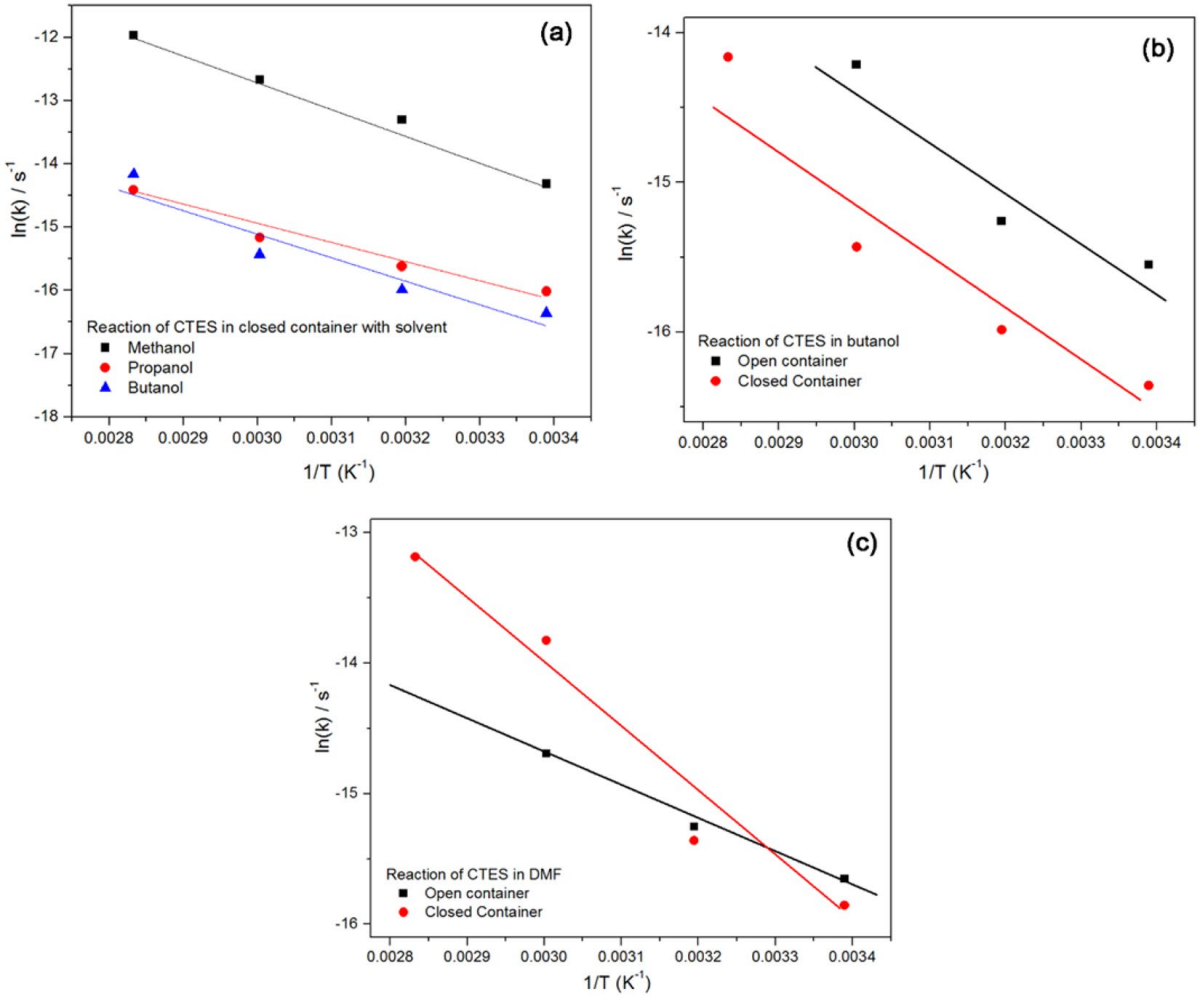
The Arrhenius plots for the CTES reaction in (**a**) alcohols in a closed system, (**b**) butanol, and (**c**) DMF in opened and closed systems.

Figure 10(a) shows the Arrhenius plots for the hydrolysis of CTES in different alcohols. The activation energies in the different alcohols were generally very close; the reaction seems to be controlled by entropy. The pre-exponential factors increased with decreasing the molecular weight, or more accurately, the molecular volume of the alcohol. This was probably due to steric effects. The Arrhenius plots of the alcohols also became more curved as the molecular weight increased.

Figure 10(b,c) show the Arrhenius plots of the CTES hydrolysis in two different solvents. Both solvents were polar, while the butanol is a protic and the DMF is an aprotic solvent. In the case of butanol the curve of the open container is almost parallel to that in the closed container, with the values for the open container larger than those for the closed container. In the case of DMF, the curve for the open container has a different slope and intercepts that for the closed container.

### Linear free energy relationships (LFER)

**Figure 11:**
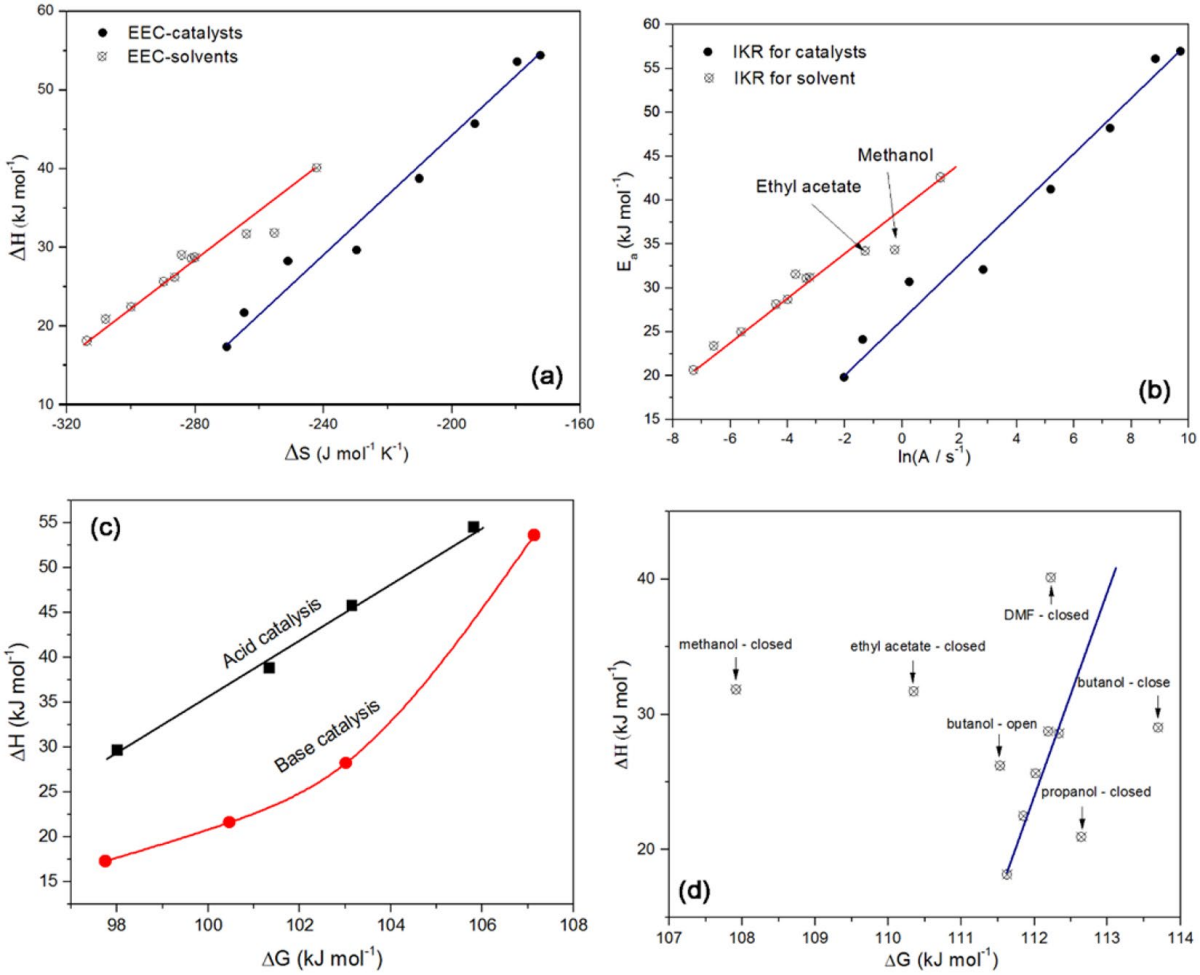
(**a**) EEC, (**b**) IKR, and relation between ∆G^ǂ^ and ∆H^ǂ^ of (**c**) catalytic reaction and (**d**) in different solvents for CTES hydrolysis in different solvents and with different catalysts (type and concentration).

The hydrolysis of CTES in different solvents, and in different catalyst concentrations in DMF, showed an enthalpy-entropy compensation (EEC) and an isokinetic relationship (IKR) that are related to extra-thermodynamics and LFER (Fig. 11(a,b), Table (6) and data in our previous work^14^). The relation between ∆H^ǂ^ and ∆S^ǂ^ is linear and is represented by the following equations: ∆*H*^ǂ^ = 269.73 ∆*S*^ǂ^ + 103.69 kJ with *R*^2^ = 0.943 and ∆*H*^ǂ^ = 366.36 ∆*S*^ǂ^ + 117.21 kJ with *R*^2^ = 0.974 for different catalyst concentrations. The relation between the activation energy (E_a_) and pre-exponential factor (A) for the reaction in different solvents and with different catalysts can respectively be described by the following equations: ${E}_{a}=2.24\,\mathrm{ln}(A)+37.87\,{\rm{kJ}}$ with R^2^ = 0.944, and ${E}_{a}=3.05\,\mathrm{ln}(A)+26.92\,{\rm{kJ}}$ with R^2^ = 0.974. The iso-kinetic temperatures for the solvents and catalysts are 270 and 366 K, respectively. Some authors called these isokinetic relationships, while others called it enthalpy-entropy compensation (EEC)^23^. The ∆G^ǂ^ of CTES hydrolysis in methanol and ethyl acetate resulted from an intermediate step which is re-esterification in the case of methanol, and hydrolysis of ethyl acetate at high temperature to produce acetic acid and ethanol, and autocatalysis by acetic acid. Some researchers claimed that these relations are artifacts^23^, and other said they are real relationships resulting from the potential-well theory. Linert^12,13^, who extensively studied the IKR, regarded the latter as real, but mentioned that the data should be carefully evaluated. He described two methods to evaluate the realness of IKR; the first one is by drawing ∆G against ∆H. If it gives a straight line, the IKR is real, otherwise the IKR is an artifact or the reaction have different mechanisms.

**Table 6:** Thermodynamic values of CTES hydrolysis reaction in different solvents.

Solvent	Container	Intercept	Slope	R^2^	E_a_ (kJ mol^−1^)	A (s^−1^)	∆H^ǂ^ (kJ mol^−1^)	∆S^ǂ^ (J K^−1^)	∆G^ǂ^ (kJ mol^−1^)
Acetonitrile	closed	−4.40	−3382	0.946	28.12	0.012	25.64	−289.84	112.02
Propanol	open	−3.35	−3736	0.998	31.07	0.035	28.59	−281.04	112.34
	closed	−6.57	−2813	0.947	23.39	0.001	20.92	−307.81	112.64
THF	closed	−5.62	−3001	0.955	24.95	0.004	22.47	−299.92	111.85
Methanol	closed	−0.25	−4126	0.987	34.31	0.777	31.83	−255.32	107.92
Isoamyl	closed	−3.23	−3755	0.792	31.22	0.040	28.74	−280.03	112.19
Ethyl acetate	closed	−1.28	−4112	0.916	34.19	0.277	31.71	−263.89	110.35
DMF	open	−7.27	−2480	0.980	20.62	0.001	18.15	−313.69	111.63
	closed	1.34	−5121	0.940	42.58	3.833	40.10	−242.05	112.23
Butanol	open	−3.99	−3448	0.803	28.67	0.019	26.20	−286.36	111.53
	closed	−3.72	−3790	0.856	31.51	0.024	29.03	−284.12	113.700

Figure 11(c,d) shows plots of ΔH against ΔG. The catalytic reactions clearly have two different mechanisms in acidic and alkaline media. In case of different solvents, the CTES hydrolysis reactions have different mechanisms, where some of the Arrhenius lines intersect, while others are parallel or behave independently. Therefore, the solvents belonging to a particular group behave similarly according to the group’s properties, and the CTES hydrolysis mechanism depends on a particular solvent group.

## Conclusions

The reactivity of AS toward the hydrolysis reaction depends on the AS nature and on the used solvent, and significantly on the catalyst. The hydrolysis reaction under spontaneous and acidic conditions in methanol is related to the partition coefficient of AS between methanol and water. In case of acetonitrile, the dielectric constant has a remarkable effect and the hydrolysis rate was enhanced with increasing the dipole moment of alkoxysilane, and there was a significant increase in fluoride acetonitrile. The hydrolysis rate in acidic dioxane deceased significantly, because the dissociation constant of acetic acid in dioxane is small. In the case of an alkaline medium, the hydrolysis rate in dioxane was better than in acetonitrile, which could be due to the solvation of anions in acetonitrile. ATES showed exceptional behavior in an acidic medium, due to the consumption of the proton generated during the protonation of the amino-group, or due to the steric effect as a result of the hindrance by protonated solvent molecules. The amino group acted as an intramolecular catalyst in the other cases. In general, the hydrolysis rate in methanol was better than in acetonitrile and dioxane due to its ability to form an H-bond. The reaction mechanism when using hydroxide involved attacking the α-hydrogen of the ethoxy group, followed by attacking of the silicon atom by water. The Taft polarity (σ*) showed the formation of negative and positive charges on AS in alkaline and acidic media. The hydrolysis reaction showed an enthalpy-entropy compensation (EEC) and isokinetic relationship (IKR).

## Supplementary information

Table S1Properties of the used solvents
