# Supplementary material for: Kinetics of alkoxysilanes hydrolysis: An empirical approach
Source: Sci Rep. 2019 Nov 26;9:17624. doi: 10.1038/s41598-019-54095-0 (PMC6879604; doi:10.1038/s41598-019-54095-0)
Supplement: Supplementary file 1 — Table S1Properties of the used solvents [file 41598_2019_54095_MOESM1_ESM.docx]

**Kinetics of alkoxysilanes hydrolysis: An empirical approach**

Ahmed A. Issa^a^, Marwa S. Elazazy ^a^, Adriaan S. Luyt^b^*

^a^ Department of Chemistry and Earth sciences, CAS, Qatar University, Doha, Qatar

^b^ Center of Advanced Materials, Qatar University, Doha, Qatar

*** Corresponding author (aluyt@qu.edu.qa)**

**Table S1 Properties of the used solvents [1,2]**

|  | **Aprotic solvents** | | | | | **Protic solvents** | | | | | |
| --- | --- | --- | --- | --- | --- | --- | --- | --- | --- | --- | --- |
|  | Polar | | Non- polar | | | Strong-----------------------------------------> Weak | | | | | Strong |
| **Property** | Acetonitrile | DMF | Dioxane | THF | Ethyl acetate | Methanol | Ethanol | Propanol | Butanol | Iso amyl | Glycerol |
| Page in the book | 309 | 265 | 233 | 237 | 247 | 81 | 85 | 89 | 97 | 113 | none |
| Molecular weight | 41 | 73 | 88 | 72 | 88 | 32 | 46 | 60 | 74 | 88 | 92.1 |
| Viscosity | 0.3409 | 0.82 | 1.3 | 0.55 | 0.46 | 0.6 | 1.08 | 1.72 | 3 | 4.2 | 934 |
| Density | 0.77 | 0.945 | 1.034 | 0.888 | 0.895 | 0.792 | 0.789 | 0.804 | 0.81 | 0.81 | 1.26 |
| Refractive index | 1.342 | 1.427 | 1.42 | 1.404 | 1.37 | 1.326 | 1.359 | 1.383 | 1.397 | 1.4014 | 1.471 |
| logP | -0.34 | -0.74 | -0.42 | 0.46 | 0.73 | -0.82 | -0.32 | 0.34 | 0.88 | 1.16 | -1.76 |
| Dielectric constant | 35.95 | 36.7 | 2.21 | 7.6 | 6.02 | 32.6 | 22.4 | 20.1 | 18.2 | 15.2 | 42.5 |
| Dipole moment | 3.92 | 3.8 | 0.4 | 1.75 | 1.7 | 1.7 | 1.7 | 1.7 | 1.66 | 1.8 | 2.7 |
| Vapor pressure | 71 | 3.8 | 32 | 133 | 78 | 103 | 45.7 | 13.4 | 4.8 | 2.4 | 1.68E-04 |
| Antoine equation  (a, b and c) | 6.999  1482  250.5 | 7.10850  1537.78  210.390 | 7.43155  1554.67 240.337 | 6.99515  1202.29  226.254 | 7.10179 1244.95 217.881 | 8.08097 1582.27 239.726 | 8.11220 1592.86 226.18 | 8.37895 1788.02 227.438 | 7.83800 1558.19 196.881 | 7.382 1373.8 174.3 |  |
| Surface tension | 29.1 | 35 | 40 | 28 | 24 | 22.6 | 22.3 | 23.7 | 24.6 | 23.8 | -- |
| Solubility in water (% w/w) | total | total | total | total | 3.3 | total | Total | total | 20.4 | ---- | total |
| Solubility parameter | 11.9 | 12.1 | 10 | 9.1 | 9.1 | 14.5 | 13.4 | 11.9 | 11.4 | -- | -- |
| Relative polarity (water) | 0.46 | 0.44 | 0.164 | 0.21 | 0.23 | 0.762 | 0.654 | 0.617 | 0.602 | 0.565 | 0.812 |
| Concentration of water (M) | 2 | 2 | 2 | 2 | 2 | 2 | 2 | 2 | 2 | 2 |  |
| Water volume (ml) | 0.36 | 0.36 | 0.36 | 0.36 | 0.36 | 0.36 | 0.36 | 0.36 | 0.36 | 0.36 |  |
| Donor number (DN) | 14.1 | 26.6 | 14.8 | 20 | 17.1 | 19 | 19.2 | 19.8 | 19.5 |  | 19 |
| Acceptor Number (AN) | 18.9 | 16 | 10.3 |  |  | 41.5 | 37.9 |  | 36.8 |  |  |
| $E_{T}^{N}$ | 0.460 | 0.386 | 0.164 | 0.207 | 0.228 | 0.790 | 0.682 | 0.617 | 0.586 | 0.565 |  |
| Solvent acidity | 0.37 | 0.30 | 0.19 | 0.17 | 0.21 | 0.75 | 0.66 | 0.63 |  |  |  |
| Solvent basicity | 0.86 | 0.93 | 0.67 | 0.67 | 0.59 | 0.5 | 0.45 | 0.44 |  |  |  |

^1^ Smallwood, I.M. *Handbook of organic solvent properties*. (Amold, a member of the Hodder Headline Group, 1996).

^2^ Cox, B.G. *Acids and Bases. Solvent Effects on Acid–Base Strength.*, (Oxford University Press, 2013).
